# Comparative evaluation of the diagnosis, reporting and investigation of malaria cases in China, 2005–2014: transition from control to elimination for the national malaria programme

**DOI:** 10.1186/s40249-016-0163-4

**Published:** 2016-06-27

**Authors:** Jun-Ling Sun, Sheng Zhou, Qi-Bin Geng, Qian Zhang, Zi-Ke Zhang, Can-Jun Zheng, Wen-Biao Hu, Archie C. A. Clements, Sheng-Jie Lai, Zhong-Jie Li

**Affiliations:** Division of Infectious Diseases, Key Laboratory of Surveillance and Early-warning on Infectious Disease, Chinese Center for Disease Control and Prevention, 155 Changbai Road, Changping District, Beijing, 102206 China; State Key Laboratory of Virology and College of Life Sciences, Wuhan University, Wuhan, 430072 China; Center of Clinical Laboratory, First Affiliated Hospital, College of Medicine, Zhejiang University, Hangzhou, China; School of Public Health and Social Work, Queensland University of Technology, Brisbane, Australia; Research School of Population Health, College of Medicine, Biology and Environment, The Australian National University, Canberra, Australia; Department of Geography and Environment, University of Southampton, Southampton, SO17 1BJ UK

**Keywords:** Malaria, Surveillance, Evaluation, Elimination, China

## Abstract

**Background:**

The elimination of malaria requires high-quality surveillance data to enable rapid detection and response to individual cases. Evaluation of the performance of a national malaria surveillance system could identify shortcomings which, if addressed, will improve the surveillance program for malaria elimination.

**Methods:**

Case-level data for the period 2005–2014 were extracted from the China National Notifiable Infectious Disease Reporting Information System and Malaria Enhanced Surveillance Information System. The occurrence of cases, accuracy and timeliness of case diagnosis, reporting and investigation, were assessed and compared between the malaria control stage (2005–2010) and elimination stage (2011–2014) in mainland China.

**Results:**

A total of 210 730 malaria cases were reported in mainland China in 2005–2014. The average annual incidence declined dramatically from 2.5 per 100 000 people at the control stage to 0.2 per 100 000 at the elimination stage, but the proportion of migrant cases increased from 9.8 % to 41.0 %. Since the initiation of the National Malaria Elimination Programme in 2010, the overall proportion of cases diagnosed by laboratory testing consistently improved, with the highest of 99.0 % in 2014. However, this proportion was significantly lower in non-endemic provinces (79.0 %) than that in endemic provinces (91.4 %) during 2011–2014. The median interval from illness onset to diagnosis was 3 days at the elimination stage, with one day earlier than that at the control stage. Since 2011, more than 99 % cases were reported within 1 day after being diagnosed, while the proportion of cases that were reported within one day after diagnosis was lowest in Tibet (37.5 %). The predominant source of cases reporting shifted from town-level hospitals at the control stage (67.9 % cases) to city-level hospitals and public health institutes at the eliminate stage (69.4 % cases). The proportion of investigation within 3 days after case reporting has improved, from 74.6 % in 2010 to 98.5 % in 2014.

**Conclusions:**

The individual case-based malaria surveillance system in China operated well during the malaria elimination stage. This ensured that malaria cases could be diagnosed, reported and timely investigated at local level. However, domestic migrants and overseas populations, as well as cases in the historically malarial non-endemic areas and hard-to-reach area are new challenges in the surveillance for malaria elimination.

**Electronic supplementary material:**

The online version of this article (doi:10.1186/s40249-016-0163-4) contains supplementary material, which is available to authorized users.

## Multilingual abstracts

Please see Additional file 1 for translations of the abstract into the six official working languages of the United Nations.

## Background

Malaria is considered one of the most significant tropical diseases of humans, being a vector borne plasmodial infection transmitted via the bites of the female *Anopheles* mosquito [1]. According to the latest global estimates from the World Health Organization (WHO), a total of 214 million cases of malaria and 438 000 deaths occurred in 2015 [2]. Significant progress has been made towards malaria control over the past decade [3–5]. As of December 2014, of the 106 countries with sustained transmission of malaria in 2000, 19 countries are in the pre-elimination or elimination phase, and seven are in the prevention of malaria reintroduction phase [2]. To achieve the goal of elimination, a sustained and well-operated malaria surveillance system is considered as a critical measure [6]. WHO launched Global Malaria Programme’s new initiative of 3T, Test, Treat, and Track in 2012, which supports malaria-endemic countries in their effort to achieve universal coverage with diagnostic testing and antimalarial treatment, as well as in strengthening malaria surveillance [7]. This program and the implementation of the 3T is contingent on the provision of timely and accurate surveillance data to monitor performance and identify threats to malaria control and elimination.

A national malaria elimination program (NMEP) was launched in China in 2010, with the goal of nationwide elimination of malaria by 2020 [8]. The elimination stage is different from the control stage, and requires monitoring and responding to each individual malaria infection, and to ultimately stop local malaria transmission [6, 9, 10]. China developed a case-based malaria surveillance system to collect information required for diagnoses and investigations, and to facilitate a rapid response to individual cases [9, 10]. For the elimination of malaria, it is essential to understand the strengths and limitations of the program by quantitatively evaluating the performance and efficiency of NMEP [11].

In this study, we compare the critical components of malaria surveillance for elimination, including diagnosis, reporting and investigation of cases between control stage and elimination stage, to evaluate the operational performance of malaria surveillance system in China, and to further improve the surveillance for malaria elimination.

## Methods

### National malaria surveillance system

In the People’s Republic of China, malaria is a notifiable infectious disease; case definitions are listed in the unified criteria issued by the Chinese National Health and Family Planning Commission [12]. It is mandatory that all suspected, probable and laboratory-confirmed cases should be reported to the malaria surveillance system. A laboratory-confirmed case is defined as a case with: malaria parasites confirmed by microscopy, a positive rapid diagnostic test (RDT), a positive polymerase chain reaction test (PCR), or a case presentation with or without typical malaria symptoms. All other cases with malaria-like symptoms and a history of travel to a malaria endemic area during malaria transmission season, or a history of blood transfusion in past 2 weeks, but without positive laboratory test results, were classified as suspected or probable cases [12]. Both of these kinds of cases were regarded as non-laboratory confirmed malaria cases in this study.

In China, the national malaria surveillance program consists of two systems: National Notifiable Infectious Disease Reporting Information System (NIDRIS) and Malaria Enhanced Surveillance Information System (MESIS), both of which were developed by the Chinese Center for Disease Control and Prevention (China CDC) [10, 13–15]. NIDRIS was established in 2004, through which 39 notifiable infectious diseases were reported. Individual case information is reported by physicians in clinics and hospitals. Reported information includes demographic information, date of onset of symptoms, date of diagnosis, date of reporting, and the reporting institute. Since the initiation of the malaria elimination stage in 2010, MESIS was developed to collect detailed epidemiological information pertaining to malaria cases to aid in malaria elimination in China. MESIS collects data on the course of case diagnosis, history of travel, date of case investigation, treatment, classification (autochthonous or imported case), and the results of case verification by city-level CDC and province-level CDC. All information in MESIS is reported by the staff of local county CDCs. Information in the MESIS and NIDRIS systems can be linked by use of unique patient identifiers.

**Fig. 1:**
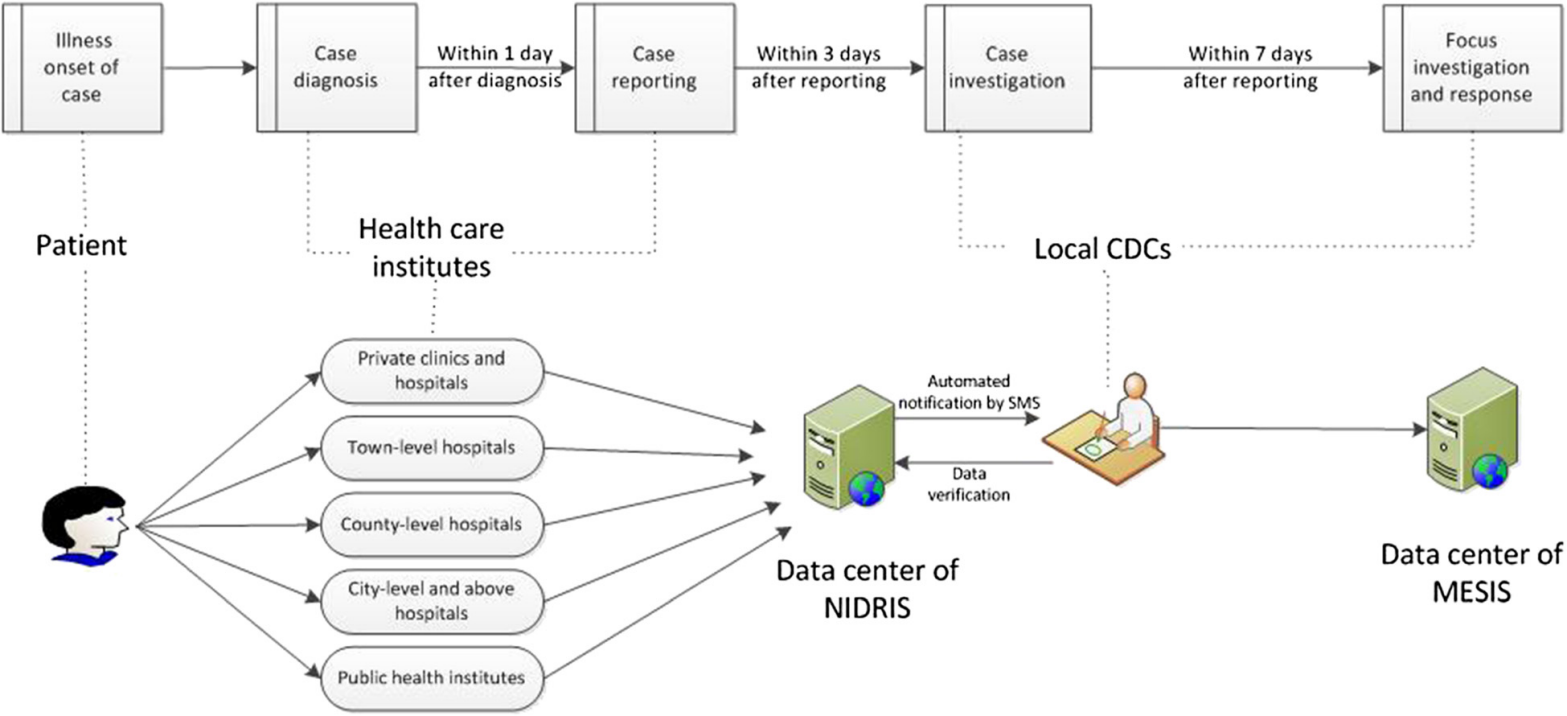
Diagram of malaria diagnosis, reporting and investigation in China (NIDRIS: National Notifiable Infectious Disease Reporting Information System; MESIS: Malaria Enhanced Surveillance Information System; CDCs: Centers for Disease Control and Prevention; SMS: Short Message Service)

The ‘1-3-7’strategy has been designed to monitor and respond to individual malaria cases during the elimination stage in China. ‘1-3-7’refers to reporting cases within 1 day after diagnosis, investigating cases within 3 days after reporting, and completing the response within 7 days after reporting [9]. The reporting requirement necessitates all hospitals and healthcare institutes across the country to report individual case information to the NIDRIS within 1 day after case diagnosis. Since 2010, if any malaria case is reported to NIDRIS, a notification is automatically sent by short text message to the staff’s cell phone in the county CDC; the staffs in the county CDC are responsible for verifying the malaria data in NIDRIS [13]. Then, staffs in the local county CDC are required to conduct an epidemiological investigation within three days after case report and to enter the investigation information into MESIS. The local area with occurrence of malaria cases would be identified as a focus, and risk assessment on local transmission would be performed by county CDC staff. Then, control measures would be taken and should be completed within seven days after reporting (Fig. 1).

### Data analysis

This study included all malaria cases recorded in NIDRIS from Jan 1, 2005, to Dec 31, 2014, and those in MESIS from Jan 1, 2011 to Dec 31, 2014. As NMEP was launched in May of 2010, the period from 2005 to 2010 was taken as the control stage, and the period from 2011 to 2014 was designated as the elimination stage in this study. The timeliness rate of reporting was calculated by dividing the number of cases reported within 1 day by the number of all reported cases. The timeliness rate of investigation was calculated by dividing the number of cases investigated within 3 days after reporting by the total number of cases investigated. The interval time from illness onset to diagnosis, diagnosis to report, and report to investigation, and the proportion of lab-confirmed cases were calculated, and comparisons between the control and the elimination stages conducted. All analyses were further stratified by the residence, locations of case, reporting hospitals or institutes, mosquito species and origin.

Each case was classified as either a local or migrant case. If the reporting institute and residential address of a case were located in the same county, the case was classified as a local case otherwise the case was classified as a migrant case. Among all 31 provinces in mainland China, the province where the case located was categorized as either an endemic or non-endemic province, and was listed in NMEP according to the historical epidemiological information of malaria in that province [8]. Non-endemic provinces included seven provinces (Beijing, Tianjin, Inner Mongolia, Heilongjiang, Jilin, Qinghai and Ningxia), the remaining 24 provinces in mainland China were regarded as endemic provinces. The reporting institutes were classified into five categories, based on their population coverage: private clinics and hospitals; town-level hospitals; county-level hospitals; city-level hospitals; and public health institutes. The city-level hospitals included the prefecture-level hospitals and provincial-level hospitals. Public health institutes covered all levels of CDCs, and Bureaus of Entry-Exit Inspection and Quarantine. Imported case was defined as patient who had a travel history to a malaria-endemic country within 1 month prior to illness onset [16]. Otherwise, the case was classified as autochthonous case.

## Results

### Occurrence of cases

A total of 210 730 malaria cases were reported during the period 2005 to 2014, with an annual average of 32 887 cases in the control stage (2005–2010), and 3 352 cases per year in the elimination stage (2011–2014). The annual incidence rate was 3.1 cases per 100 000 in 2005 and 4.7 per 100 000 in 2006, then decreased each year until 2012 (0.2 cases per 100 000 people), rose to 0.3 cases per 100 000 people in 2013. The average incidence from 2005 to 2010 was 2.5 cases per 100 000 people, this rate was more ten times than that of the period from 2011 to 2014 (0.2 cases per 100 000 people; Table 1). The majority of cases during the control stage were attributed to *P. vivax* infection (78.4 %), while *P. falciparum* infection predominated during the elimination stage (55.7 %), increasing from 9.4 % in 2005 to 63.1 % in 2014.

**Table 1:** Characteristics of malaria cases at control stage and elimination stage in China

Characteristics	Control stage (2005–2010)							Elimination stage (2011–2014)				
	2005	2006	2007	2008	2009	2010	Average per year	2011	2012	2013	2014	Average per year
Number of cases	40,226	61,204	47,380	26,727	14,278	7506	32,887	4127	2453	3905	2924	3352
Incidence rate (per 100 000)	3.1	4.7	3.6	2.0	1.1	0.6	2.5	0.3	0.2	0.3	0.2	0.2
Cases by species												
*P. vivax*	30,692	48,169	38,768	21,322	10,747	4956	25,776	2432	1021	897	851	5201
(%)	(76.3)	(78.7)	(81.8)	(79.8)	(75.3)	(66.0)	(78.4)	(58.9)	(41.6)	(23.0)	(29.1)	(38.8)
*P. falciparum*	3771	2872	1691	1025	1043	1304	1951	1442	1354	2825	1844	7465
(%)	(9.4)	(4.7)	(3.6)	(3.8)	(7.3)	(17.4)	(5.9)	(35.0)	(55.2)	(72.3)	(63.1)	(55.7)
Others	5763	10,163	6921	4380	2488	1246	5160	253	78	183	229	743
(%)	(14.3)	(16.6)	(14.6)	(16.4)	(17.3)	(16.6)	(15.7)	(6.1)	(3.2)	(4.7)	(7.8)	(5.5)
Cases by residence												
Local	36,009	55,999	43,489	24,180	12,413	5849	29,657	2639	1325	2413	1538	1979
(%)	(89.5)	(91.5)	(91.8)	(90.5)	(86.9)	(77.9)	(90.2)	(63.9)	(54.0)	(61.8)	(52.6)	(59.0)
Migrant	4217	5205	3891	2547	1865	1657	3230	1488	1128	1492	1386	1373
(%)	(10.5)	(8.5)	(8.2)	(9.5)	(13.1)	(22.1)	(9.8)	(36.1)	(46.0)	(38.2)	(47.4)	(41.0)
Cases by location^a^												
24 endemic provinces	40,182	61,130	47,312	26,659	14,221	7436	32,823	4033	2386	3801	2837	3264
(%)	(99.9)	(99.9)	(99.7)	(99.8)	(99.6)	(99.1)	(99.8)	(97.7)	(97.3)	(97.3)	(97.0)	(97.4)
7 non-endemic provinces	44	74	68	68	57	70	64	94	67	104	87	88
(%)	(0.1)	(0.1)	(0.3)	(0.2)	(0.4)	(0.9)	(0.2)	(2.3)	(2.7)	(2.7)	(3.0)	(2.6)
Cases by reporting institutes												
Private clinics and hospitals	321	334	540	347	193	182	320	62	41	69	57	57
(%)	(0.8)	(0.6)	(1.1)	(1.3)	(1.3)	(2.4)	(1.0)	(1.5)	(1.7)	(1.8)	(2.0)	(1.7)
Town-level hospitals	24,433	44,923	34,466	17,870	8767	3449	22,318	1379	298	174	129	495
(%)	(60.7)	(73.4)	(72.7)	(66.8)	(61.4)	(46.0)	(67.9)	(33.4)	(12.2)	(4.4)	(4.4)	(14.8)
County-level hospitals	5400	5764	4919	3278	1742	865	3661	403	327	711	448	472
(%)	(13.4)	(9.4)	(10.4)	(12.3)	(12.2)	(11.5)	(11.1)	(9.8)	(13.3)	(18.2)	(15.3)	(14.1)
City-level hospitals	2562	2878	2490	1840	1352	1333	2076	1186	923	1342	1363	1204
(%)	(6.4)	(4.7)	(5.3)	(6.9)	(9.5)	(17.8)	(6.3)	(28.7)	(37.6)	(34.4)	(46.6)	(35.9)
Public health institutes	7510	7305	4965	3392	2224	1677	4512	1097	864	1609	927	1124
(%)	(18.7)	(11.9)	(10.5)	(12.7)	(15.6)	(22.3)	(13.7)	(26.6)	(35.2)	(41.2)	(31.7)	(33.5)
Cases by origin^b^												
Autochthonous cases	–	–	–	–	–	–	–	1507	253	92	67	480
(%)	–	–	–	–	–	–	–	(36.5)	(10.3)	(2.4)	(2.3)	(14.3)
Imported cases	–	–	–	–	–	–	–	2620	2200	3813	2857	2872
(%)	–	–	–	–	–	–	–	(63.5)	(89.7)	(97.6)	(97.7)	(85.7)

^a^Endemic provinces include Liaoning, Xinjiang, Hebei, Shanxi, Shaanxi, Shandong, Henan, Jiangsu, Shanghai, Zhejiang, Anhui, Hubei, Hunan, Jiangxi, Fujian, Guangdong, Hainan, Guangxi, Yunnan, Sichuan, Guizhou, Chongqing, Tibet, and Gansu; non-endemic provinces include Beijing, Tianjin, Inner Mongolia, Heilongjiang, Jilin, Qinghai, Ningxia, according to the national malaria elimination program [3]

^b^Only the origins of cases occurring within the period 2011–2014 were included in this study, because each malaria case was required to be classified as either autochthonous or imported since 2010, according to the national malaria elimination program [3]

Migrant cases accounted for only 10.5 % of cases in 2005; these increased to 47.4 % in 2014. Overall, the proportion of migrant cases at the elimination stage (41.0 %) was significantly higher than that at the control stage (9.8 %; *X*^2^ = 11699.8, *P* < 0.001). Geographically, cases predominately occurred in the 24 historically endemic-provinces (99.7 %). The proportion of cases occurring in the seven non-endemic provinces increased over the course of the study, from 0.1 % in 2005 to 3.0 % in 2014. During the elimination stage, the proportion of autochthonous cases declined dramatically, from 36.5 % in 2011 to 2.3 % in 2014. The proportion of cases reported by town-level hospitals reduced sharply from 67.9 % during the control period to only 14.8 % in the elimination stage. In contrast, during the period from 2011 to 2014, city-level hospitals and public health institutes became the predominant sources of case reporting (67.4 %). The proportion of cases reported by both city-level hospitals and public health institutes showed an increasing trend, from 28.7 % in 2011 to 46.6 % in 2014, and from 26.6 % in 2011 to 31.7 % in 2014, respectively.

### Case diagnosis

**Fig. 2:**
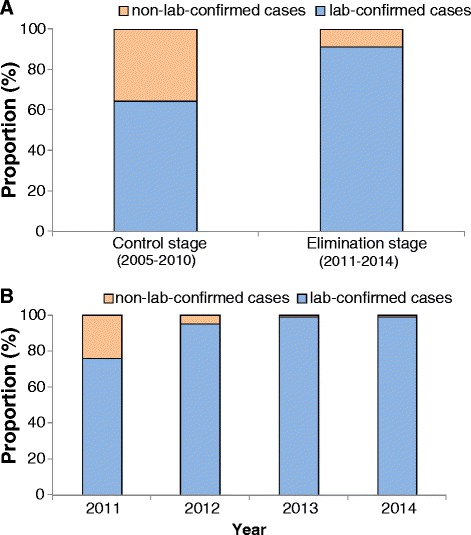
Proportion of lab-confirmed malaria cases during 2005–2014 in China (**a** proportion of cases between control stage and elimination stage; **b** proportion of cases by year during elimination stage)

**Fig. 3:**
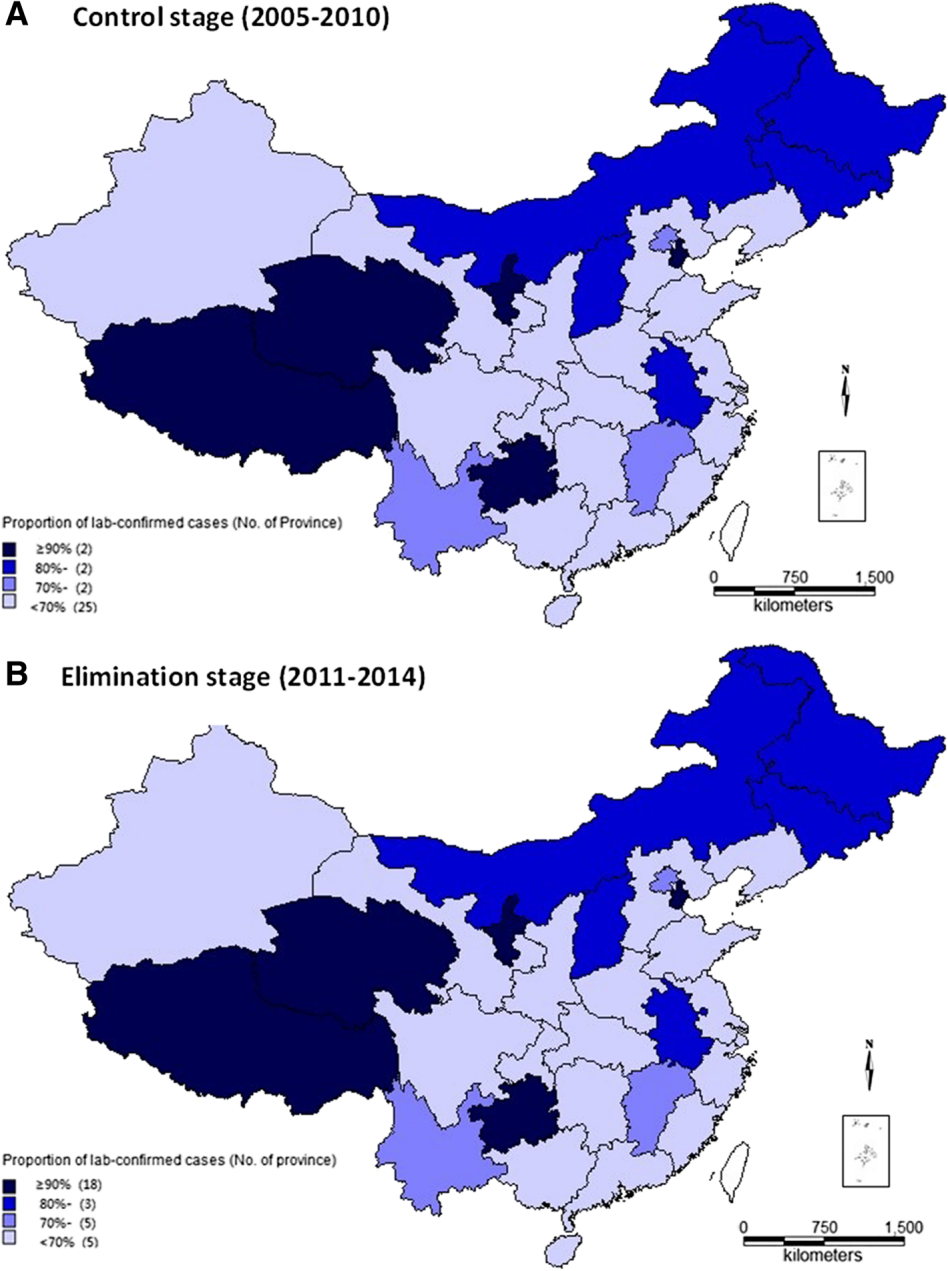
Proportion of lab-confirmed malaria by province during 2005–2014 in China (**a** control stage [2005–2010]; **b** elimination stage [2011–2014])

The majority of malaria cases (66.2 %; 139 498) were laboratory confirmed. The proportion of cases that were laboratory confirmed (P_Lab_) in the elimination stage (91.1 %) was higher than for the control stage (64.5 %; Fig. 2a). Furthermore, during the elimination stage, P_Lab_ showed an increasing yearly trend, from 75.9 % in 2011 to 99.0 % in 2014 (Fig. 2b). P_Lab_ in malaria-endemic provinces (91.4 %) was significantly higher than that in the non-endemic provinces (79.0 %; *X*^2^ = 65.8, *P* < 0.001) for the period 2011 to 2014. The number of provinces with P_Lab_ greater than 90 % increased from two provinces (2005 and 2010) to 18 provinces (2011 to 2014). The seven non-endemic provinces were among the 13 provinces with P_Lab_ lower than 90 % during 2011–2014 (Fig. 3).

There were five provinces with P_Lab_ < 70 % during 2011–2014: Tibet (8/24, 33.3 %), Guizhou (72/211, 34.1 %), Ningxia (8/14, 57.1 %), Tianjin (32/48, 66.7 %) and Qinghai (9/13, 69.2 %). The average P_Lab_ of these five provinces was 14.0 % in 2011 and 54.1 % in 2012, improved markedly in 2013 and 2014 (87.5 % and 89.1 %, respectively). During the period 2011 to 2014, 114 cases were reported by town-level hospitals in five provinces and their P_Lab_ (9/114) was 7.9 % which was lower than that from other reporting institutes (120/196, 61.2 %). Among the 105 non-laboratory-confirmed cases, 104 cases were from Guizhou Province.

**Fig. 4:**
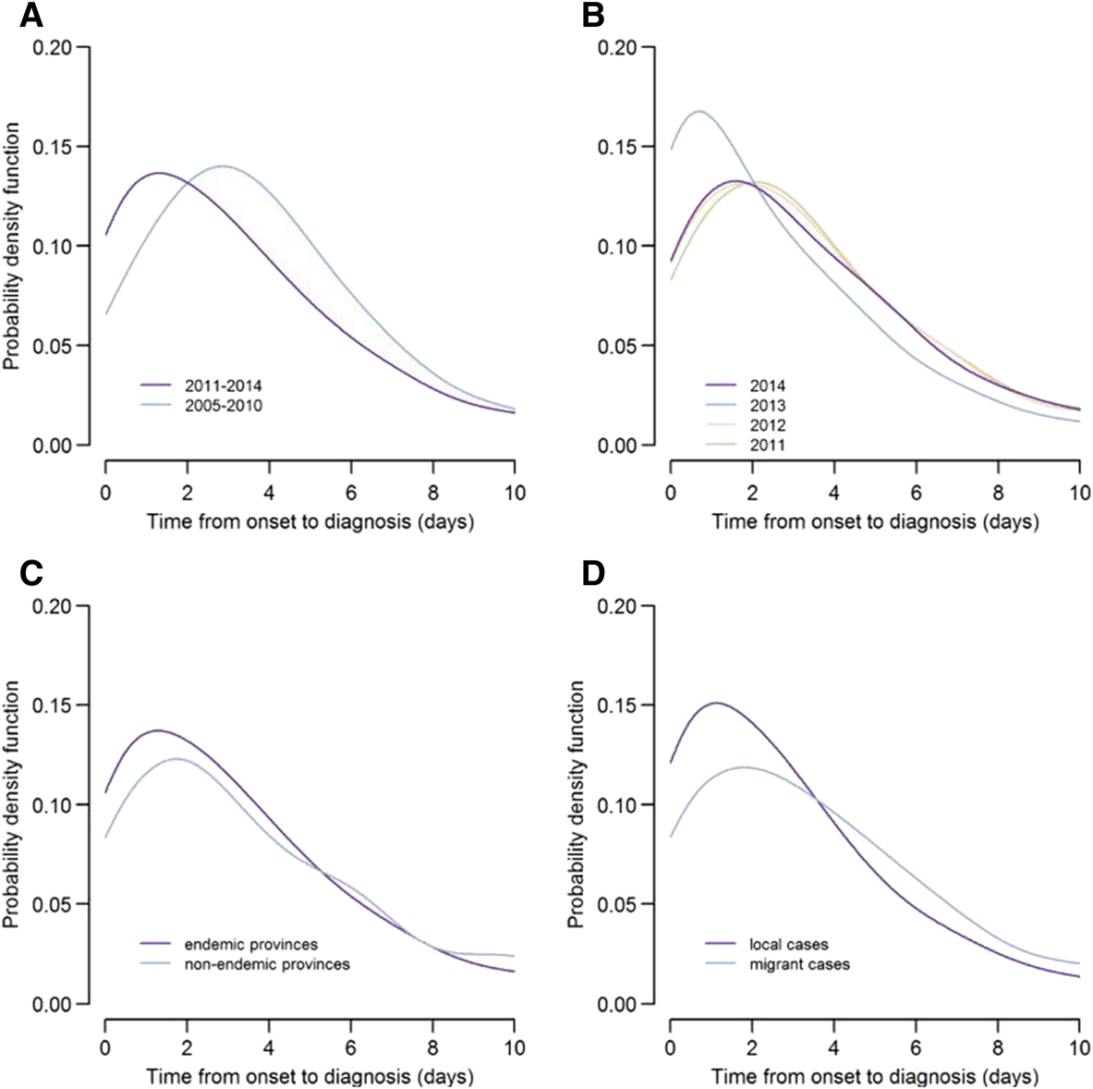
Distributions of time from illness onset to diagnosis of malaria cases in China (**a** by stage; **b** by year; **c** by case geographical distribution of cases during 2011–2014; **d** by case migration during 2011–2014)

During 2005–2014, the median interval from onset of illness to diagnosis (T_diag_) was 4 days (IQR: 2–6). The median T_diag_ was 3 days during the elimination stage (IQR: 1–6), one day earlier than the control stage (Fig. 4a).

The timeliness of diagnosis was stable during the period from 2011 to 2013, with the median T_diag_ being 2 days in 2013, and 3 days during the other 3 years (Fig. 4b). There was no difference of median T_diag_ between *P. vivax* and *P. falciparum* cases during the period 2011 to 2014. The proportion of cases with T_diag_ < 4 days was higher for endemic provinces (65.0 %) than for non-endemic provinces (56.5 %; *X*^2^ = 10.7, *P* = 0.001). The proportion of cases with T_diag_ < 4 days was also higher for local cases (69.9 %) than migrant cases (57.3 %; *X*^2^ = 224.7, *P* < 0.001; Fig. 4c and d). The reporting institutes were associated with T_diag_, where the proportion of cases reported from city and higher-level hospitals with T_diag_ ≤4 days (58.9 %) was lower than that from other hospitals or public health institutes (70.9 %) (*X*^2^ = 209.1, *P* < 0.001).

### Case reports

For the period 2005 to 2014, 86.1 % cases were reported within one day after diagnosed. There was a significant increase in the proportion of notifications within one day over the course of the study, from 85.2 % at the control stage to 99.7 % in elimination stage (*X*^2^ = 2115.0, *P* < 0.001). From 2011, more than 99 % of cases were reported within 24 h after diagnosis (Table 2). From 2011 to 2014, reporting of 40 cases occurred more than 1 day after diagnosis; these came from 16 provinces; most occurred in Tibet (15 cases) and Anhui province (6 cases). The proportion of cases that were reported within one day after diagnosis was lowest in Tibet (37.5 %) from 2011 to 2014. Reporting rates less than a day (from diagnosis) was over 95 % in all the other provinces. Of the 40 instances of delayed reporting, 50 % (20 cases) were reported by public health institutes and 35 % (14 cases) were reported by city-level hospitals.

**Table 2:** The reports and investigations for malaria cases in China, 2011–2014

Item	2011	2012	2013	2014	Total
Number of case reports	4094	2433	3793	2519	12,839
Median interval time from diagnosis to report (day)	0	0	0	0	0
IQR of interval time from diagnosis to report (day)	0–1	0–1	0–1	0–1	0–1
Percentage of cases reported within 1 day after diagnosis (%)	99.9	99.5	99.8	99.4	99.7
Number of case investigations	3293	2007	3203	2564	11,067
Median interval time from report to investigation (day)	1	0	0	0	0
IQR of interval time from report to investigation (day)	0–4	0–2	0–1	0–1	0–1
Percentage of investigations made within 3 days after reports (%)	74.6	88.6	99.3	98.5	89.9

### Case investigations

During the period August 2012 to December 2014, there were 14,295 short messages alerts automatically sent to the staffs of local CDCs; 99.0 % of which were acted upon appropriately. The median interval from the alert to data verification was 0.84 h (IQR: 0.18–4.4 h); 65.0 % of alerts were verified by the local CDCs within 2 h of the message being sent.

During the malaria elimination stage (2011–2014), the median interval from report to investigation was less than one day (IQR: 0–2 days). For the period 2011 to 2014, 89.9 % (9 944/11 067) of cases were investigated within 3 days after case reported, with an increasing trend from 74.6 % in 2010 to 98.5 % in 2014 (Table 2). The timeliness of case investigation for 2013–2014 (99.0 %) was higher than for 2011–2012 (80.1 %). Amongst migrant cases, 9.2 % (418/4 549) cases were not investigated within three days of reporting during 2011 to 2014, which was slightly lower than that for local cases (10.8 %, 705/6 518, *X*^2^ = 7.8, *P* = 0.005). There was no significant difference in the proportion of cases investigated within 3 days between endemic and non-endemic provinces (*X*^2^ = 0.89, *P* = 0.35).

## Discussion

Over the past 10 years, with the effective control of the malaria epidemic, China has shifted it approach to malaria from control (2005–2010) to an elimination phase (2011–2014) [17–22]. This study described changes in the source of malaria cases over this period and reports on the accuracy and timeliness of malaria diagnosis, reporting and investigation.

One of the key features of a malaria surveillance system, in the elimination phase, is to be sufficiently sensitive to detect all malaria infections and facilitate early treatment to prevent secondary cases [3, 6]. All suspected cases of malaria should receive a parasitological test at the elimination stage [16]. This study demonstrated that the proportion of laboratory-confirmed cases has increased significantly from the control stage (64.5 %) to the elimination stage (91.1 %) adding diagnostic certainty to existing epidemiological data. This finding concurs with the findings of other studies [23]. A study of the diagnosis and reporting of malaria cases in China during 2005–2008 concluded that the capacity for laboratory diagnosis needed to be further strengthened, especially in the local medical institutes [24]. This appears to have occurred in the majority of provinces, however, P_Lab_ was still <70 % in five provinces from 2011–2014. Furthermore, the P_Lab_ in the town-level hospitals (7.9 %) was lower than that for other reporting institutes. This indicates that the capacity for diagnosis in town-level hospitals remains a challenge for malaria elimination programmes, especially in the five provinces with low capacity of laboratory testing. The proportion of laboratory-confirmed cases occurring in non-endemic provinces was also found to be significantly lower than for endemic provinces. This may be a function of a lack of perceived risk, experience, skills or due to poor personal training in the facilities of hospitals and CDCs in these regions; regardless, this represents another area that needs to be strengthened.

Timeliness is one of important attributes of surveillance system evaluation [25, 26]. In this study, the timeliness of case reporting has maintained at higher proportions (above 99 %) during 2011 to 2014, which might indicate the improvement of the internet-based reporting approach of NIDRIS. However, our analysis found that the P_Lab_ and the proportion of cases reported in 1 day was the lowest in Tibet during 2011 to 2014 due to the poor capacity of malaria control and prevention in the local CDC of Tibet, and the low accessibility of healthcare services by inconvenient transport [27, 28]. With an aim to achieve a nationwide elimination goal, China’s “1-3-7” surveillance and response strategy should also supported and focus on those remote areas with malaria reported [29].

There are a number of challenges that need to be addressed if China is to successfully eliminate malaria. Population movement has the potential to spread malaria from endemic areas to non-endemic areas or to reintroduce malaria to regions where it has been eliminated [30]. In our study, one apparent characteristic of malaria cases was the increasing proportion of migrant cases in the elimination phase; the need to address this issue has been identified by other researchers [31]. The time from onset to diagnosis for migrant cases was longer than that for local cases, and the timeliness of migrant case investigation was relatively poor. These features might be associated with the characteristics of migrant population, including living in the areas with high malaria endemicity or high risk season, poor accommodation without suitable vector prevention, lack of knowledge and awareness of malaria prevention, poor awareness to seek treatment, and limited accessibility of healthcare service [32, 33]. Equitable provision of diagnosis and treatment, as well as investigation services for this population, are challenges should be taken into account during elimination stage [34]. It is imperative that the issues affecting migrant populations are addressed in national programme, such as through active screening of returning workers especially in areas with high risk of local transmission, if malaria eradication is to be achieved [31].

There were some limitations to this study. Complete data on foci investigation in MESIS during 2011 to 2014 was not available and, as such, we could not analyze the performance of foci investigation and disposal within 7 days. According to the new surveillance project issued by China CDC [35], the data on foci investigation will be collected in MESIS from 2015 onwards. This will allow the whole profile of Chinese “1-3-7” malaria strategy to be better analyzed. Moreover, only the accuracy and timeliness of reporting of surveillance system were evaluated in this study, the other attributes such as the data completeness and validity, and sensitivity and specificity for the malaria surveillance system need to be further assessed in the future.

## Conclusions

Our study found that the source of malaria case detection has changed greatly from the control to elimination stage, and the individual case-based malaria surveillance system in China generally operated well during the malaria elimination stage. However, China still faces many challenges, including the changing epidemiology of malaria cases among the domestic migrant and foreign populations, and those who are in the historically non-endemic areas, and hard-to-reach populations.

## Abbreviations

CDC, Center for Disease Control and Prevention; IQR, Interquartile Range; MESIS, Malaria Enhanced Surveillance Information System; NIDRIS, National Notifiable Infectious Disease Reporting Information System; NMEP, National Malaria Elimination Programme; PCR, Polymerase Chain reaction Test; P_Lab_, Proportion of Lab-confirmed cases; RDTs, Rapid Diagnostic Tests; T_diag_, the median time from illness onset to diagnosis; WHO, World Health Organization

## Additional file

Additional file 1: Multilingual abstracts in the six official working languages of the United Nations. (PDF 258 kb)
