# Erratum to: Comparative evaluation of the diagnosis, reporting and investigation of malaria cases in China, 2005–2014: transition from control to elimination for the national malaria programme

**DOI:** 10.1186/s40249-017-0317-z

**Published:** 2017-06-19

**Authors:** Jun-Ling Sun, Sheng Zhou, Qi-Bin Geng, Qian Zhang, Zi-Ke Zhang, Can-Jun Zheng, Wen-Biao Hu, Archie C.A. Clements, Sheng-Jie Lai, Zhong-Jie Li

**Affiliations:** 1 Division of Infectious Diseases, Key Laboratory of Surveillance and Early-warning on Infectious Disease, Chinese Center for Disease Control and Prevention, 155 Changbai Road, Changping District, Beijing, 102206 China; 2 State Key Laboratory of Virology and College of Life Sciences, Wuhan University, Wuhan, 430072 China; 3 Center of Clinical Laboratory, First Affiliated Hospital, College of Medicine, Zhejiang University, Hangzhou, China; 4 School of Public Health and Social Work, Queensland University of Technology, Brisbane, Australia; 5 Research School of Population Health, College of Medicine, Biology and Environment, The Australian National University, Canberra, Australia; 6 Department of Geography and Environment, University of Southampton, Southampton, SO17 1BJ UK

## Erratum

After publication of this article [1] it was noticed that the wrong figure was used for Fig. 3. Please see the correct Fig. 3 below.

**Fig. 3:**
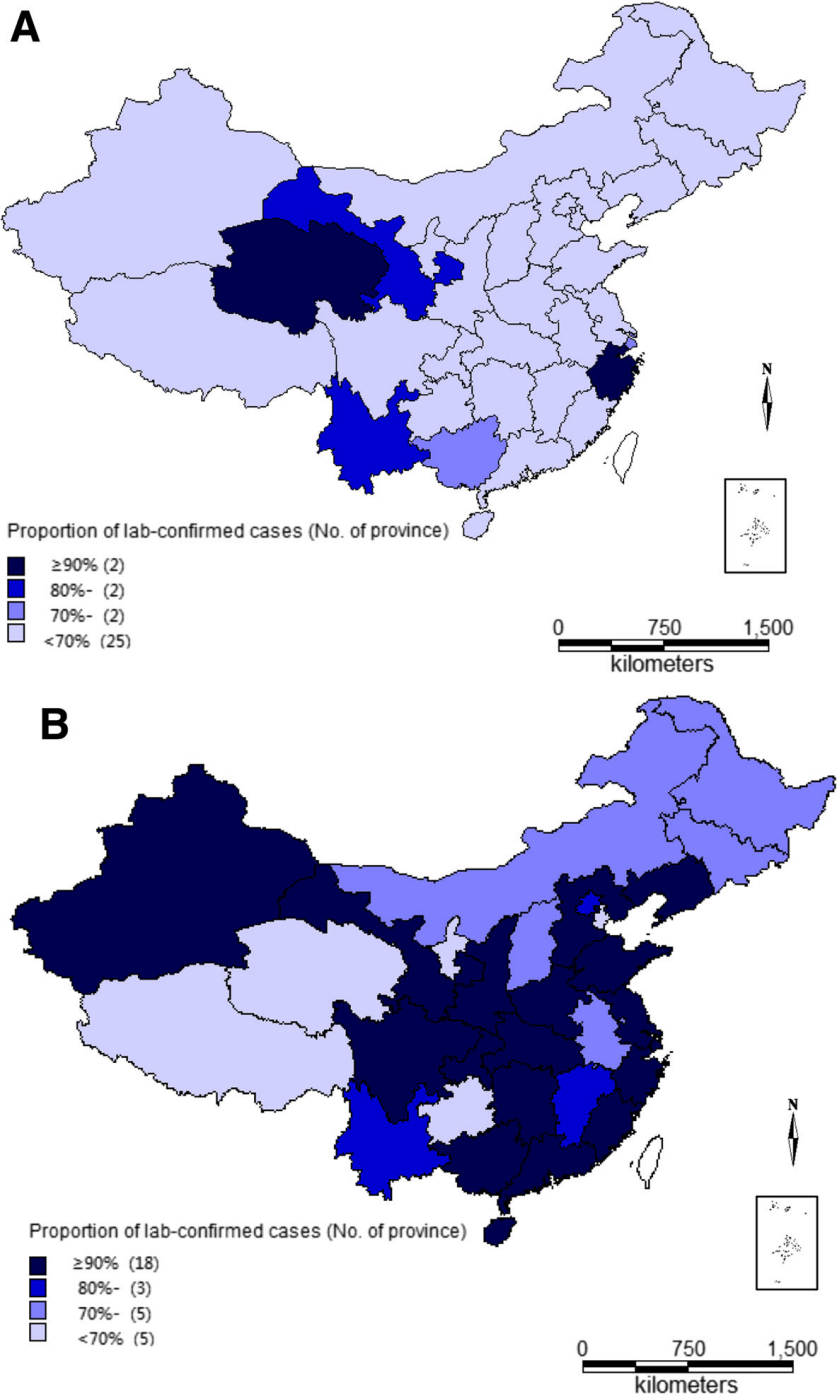
Proportion of lab-confirmed malaria by province during 2005–2014 in China (**a** control stage [2005–2010]; **b** elimination stage [2011–2014])]
