# Supplementary material for: Comparative evaluation of the diagnosis, reporting and investigation of malaria cases in China, 2005–2014: transition from control to elimination for the national malaria programme
Source: Infect Dis Poverty. 2016 Jun 27;5:65. doi: 10.1186/s40249-016-0163-4 (PMC4924285; doi:10.1186/s40249-016-0163-4)
Supplement: Additional file 1: — Multilingual abstracts in the six official working languages of the United Nations. (PDF 258 kb) [file 40249_2016_163_MOESM1_ESM.pdf]

Translation of the abstract into the six official working languages of the United Nations

تقييم مقارن لتشخيص، التقرير عن، والتحقق من حالات الاصابة بالملاريا في الصين، 2014-2005: الانتقال ببرنامج الملاريا الوطني من السيطرة على المرض الى القضاء عليه

جون-لنغ سَن، شينغ زَو، كي-بن غينج، كيان زانغ، زي-كي زانغ، كان-جون زينغ، وين-بياو هو، ارشي ك. ا. كليمنتس، شينغ-جي لاي، زونغ-جي لي

خلاصة

خلفية: يتطلب القضاء على الملاريا بيانات مراقبة ذات نوعية عالية تمكّن التحديد السريع والاستجابة العاجلة للحالات المفردة. يمكن لتقييم أداء نظام مراقبة الملاريا الوطني تعيين مواطن القصور والتي، ان تمّ التعامل معها، ستُحسّن برنامج المراقبة للقضاء على الملاريا.

الطرائق: تم استخلاص بيانات الحالات للفترة 2014-2005 من النظام الوطني الصيني لتقديم المعلومات عن الأمراض المعدية واجبة التبليغ ونظام المعلومات المعزّز لمراقبة الملاريا. وقد تم تقييم حالات الاصابة، ودقة الانضباط الزمني لتشخيص الحالات، والتقرير والابلاغ عنها؛ ومقارنتها بين مرحلتي السيطرة (2010-2005) والقضاء على المرض (2014-2011) في أنحاء الصين.

النتائج: تم الابلاغ عن 210,730 حالة اصابة بالملاريا في الصين بين 2005 و 2014. وقد هبط معدّل الاصابة السنوي بصورة كبيرة من 2.5 لكل 100,000 شخص في مرحلة السيطرة الى 0.2 لكل 100,000 شخص في مرحلة القضاء على المرض، ولكنّ نسبة الحالات المهاجرة ازدادت من 9.8% الى 41.0% منذ بدء البرنامج الوطني للقضاء على الملاريا في 2010. لقد تحسّنت النسبة الكلية للحالات المشخّصة بالفحص المختبري بصورة مستمرة، بلغت في أعلاها 99.0% عام 2014. ولكن كانت هذه النسبة أقلّ بكثير في المناطق غير المستوطنة بالمرض (79.0%) منها في المناطق المستوطنة بالمرض (91.4%) بين عامي 2011-2014. كانت متوسط الفترة بين ظهور المرض والتشخيص 3 ايام في مرحلة القضاء على المرض، وأقل من ذلك بيوم في مرحلة السيطرة على المرض. منذ 2011، تم الإبلاغ عن أكثر من 99% من الحالات خلال يوم واحد بعد التشخيص، فيما كانت نسبة الحالات التي تم الابلاغ عنها خلال يوم واحد بعد التشخيص هي الأقل في التبت (37.5%). إن المصدر السائد للحالات المبلغ عنها في المستشفيات القروية في مرحلة السيطرة على المرض (67.9% حالة) ارتفع في مستشفيات المدينة ومؤسسات الصحة العامة في مرحلة القضاء على المرض (69.4% حالة). وقد ارتفعت نسبة التحقق من الحالات المبلغ عنها خلال 3 أيام، من 74.6% في 2010 الى 98.5% في 2014.

الاستنتاجات: لقد عمِل نظام مراقبة الحالات المفردة للملاريا في الصين بصورة جيدة خلال مرحلة القضاء على المرض. أكّد ذلك على أنّ حالات الاصابة بالملاريا يمكن تشخيصها، والابلاغ عنها، والتحقق منها في وقت مناسب على المستوى المحلي. ولكن، تبقى الهجرة المحلية والعالمية، والحالات في المناطق الغير مستوطنة تاريخيا بالملاريا والمناطقة النائية تحديات تواجه مراقبة الملاريا من أجل القضاء على المرض.

Translated from English version into Arabic by Muther J. Alohmayed, through

TRANSLATORS
WITHOUT BORDERS

2005-2014 年中国疟疾病例诊断、报告和调查的定量评估：从疟疾控制至消除阶段的变化

孙军玲，周升，耿启斌，张倩，张子科，郑灿军，胡文彪，Archie C.A. Clements,赖胜杰，李中杰

摘要

引言: 实现国家疟疾消除策略要求高质量的监测数据，从而快速识别和调查处置每一例疟疾病例。通过对全国疟疾监测系统运行情况开展定量评估，有利于提高消除阶段疟疾监测系统的灵敏性。

方法:从全国法定报告传染病信息报告系统和疟疾专病监测信息系统中收集 2005-2014 年疟疾病例的个案数据。对疟疾控制阶段（2005-2010 年）和消除阶段（2011-2014 年）中国大陆疟疾病例的病例诊断、报告和个案调查的准确性和及时性进行评估和比较。

结果: 2005-2014 年中国大陆共报告 210 730 例疟疾病例。从控制阶段至消除阶段，疟疾的平均年发病率由 2.5/100 000 下降至 0.2/100 000，而流动人口病例的比例由 9.8%上升至 41.0%。自 2010 年启动全国疟疾消除行动计划以来（消除阶段），实验室诊断病例的比例持续提高，在 2014 年达到最高（99.0%），但非流行区省份实验室诊断病例的比例（79.0%）显著低于流行区省份（91.4%）。消除阶段疟疾病例从发病至诊断的时间间隔中位数为 3 天，比控制阶段早 1 天。自 2011 年以来，99%以上的疟疾病例均在诊断 1 天内报告，但西藏病例在诊断 1 天以内报告的比例最低（37.5%）。病例报告的主要单位由控制阶段的乡镇卫生院（占 67.9%），转变为消除阶段的市级及以上医院和公共卫生机构（占 69.4%）。病例报告后 3 天内完成个案调查的比例也在逐年升高，由 2010 年的 74.6%上升至 2014 年的 98.5%。

结论: 在疟疾消除阶段，中国以病例个案为基础的全国疟疾监测系统运行良好，这对确保疟疾病例在当地能得到及时的诊断、报告及个案调查。然而，国内外流动人口、非既往流行区、卫生服务可及性差的地区的病例将是消除阶段疟疾监测的巨大挑战。

Translated from English version into Chinese by Sun Jun-ling and Li Zhong-Jie

Évaluation comparative du diagnostic, du signalement et de la recherche de cas de paludisme en Chine entre 2005 et 2014 : transition du contrôle vers l'élimination dans le programme national de lutte contre le paludisme

Jun-Ling Sun, Sheng Zhou, Qi-Bin Geng, Qian Zhang, Zi-Ke Zhang, Can-Jun Zheng, Wen-Biao Hu, Archie C.A. Clements, Sheng-Jie Lai and Zhong-Jie Li

Résumé
Contexte: L'élimination du paludisme requiert des données de surveillance de très bonne qualité pour permettre une détection des cas et une réponse rapides. L'évaluation des performances d'un système national de surveillance du paludisme peut identifier les défauts dont la rectification apportera une amélioration au programme de surveillance pour l'élimination du paludisme.
Méthodes : Des données de cas ont été extraites pour la période de 2005 à 2014 du Système d'informations pour le signalement des maladies infectieuses à déclaration obligatoire chinois et du Système d'informations pour la surveillance du paludisme renforcée. La prévalence des cas, la précision et la pertinence dans le temps des diagnostics, du signalement et de l'investigation ont été évaluées et comparées entre le stade de contrôle du paludisme (2005-2010) et le stade d'élimination (2011-2014) en Chine continentale.
Résultats: 210 730 cas de paludisme ont été signalés en R.P. de Chine entre 2005 et 2014. L'incidence moyenne annuelle a diminué considérablement, de 2,5 pour 100 000 pendant la phase de contrôle à 0,2 pour 100 000 au stade d'élimination, mais la proportion de cas migrants a augmenté de 9,8 % à 41,0 %. Depuis le lancement du programme national d'élimination du paludisme en 2010, la proportion globale de cas diagnostiqués par des tests de laboratoire a significativement augmenté, atteignant un maximum de 99,0 % en 2014. Cependant, cette proportion est significativement plus faible dans les provinces où le paludisme n'est pas endémique (79,0 %) que dans les provinces d'endémie (91,4 %) sur la période de 2011 à 2014. L'intervalle médian entre le début de la maladie et le diagnostic était de 3 jours pendant la phase d'élimination, soit un jour de moins que pendant la phase de contrôle. Depuis 2011, plus de 99 % des cas ont été signalés moins d'un jour après avoir été diagnostiqués, la proportion la plus basse de cas signalés un jour après le diagnostic se rencontrant au Tibet (37,5 %). La source prédominante de signalement des cas n'est plus les hôpitaux locaux comme pendant la phase de contrôle (67,9 % des cas) mais les centres hospitaliers des grandes villes et les établissements de santé publics pendant la phase d'élimination (69,4 % des cas). La proportion des investigations menées dans les trois jours suivant le signalement a augmenté de 74,6 % en 2010 à 98,5 % en 2014.
Conclusions: Le système chinois de surveillance du paludisme basé sur les cas individuels a bien fonctionné pendant la phase d'élimination du paludisme. Les cas de paludisme ont ainsi pu diagnostiqués, signalés et analysés en temps utile au niveau local. Toutefois, les migrations entre provinces et de l'étranger constituent un nouveau défi pour la surveillance en vue de l'élimination du paludisme, tout comme les cas qui surviennent dans les zones historiquement exempts d'endémie et les régions difficiles d'accès.

Translated from English version into French by Suzanne Assenat, through

TRANSLATORS
WITHOUT BORDERS

Сравнительная оценка диагноза при учете и исследовании случаев заболевания малярией в Китае в 2005–2014: переход от контроля к ликвидации в рамках национальной программы борьбы с малярией

Джун-Лин Сум, Шенг Джу, Чи-Бин Ген, Чян Джанг, Дзи-Ке Джанг, Цан-Дзун Дженг, Вен-Бяо Ху, Арчи С.А. Клементс, Шенг-Джи Лай и Джонг-Дже Ли

Резюме
Обоснование: Ликвидация малярии требует данных наблюдения высокого качества для обеспечения быстрого обнаружения и реагирования на отдельные случаи. Оценка выполнения национальной системы эпиднадзора за малярией может выявить недостатки, уведомление о которых улучшит программу борьбы с малярией.
Методы: Данные за период 2005–2014 были извлечены из Китайской национальной системы регистрации инфекционных заболеваний информационной системы и Системы усиленного контроля малярии. Возникновение случаев, точность и своевременность диагнозов, отчетности и расследования, оценивались и сравнивались между стадией борьбы с малярией (2005-2010 годы) и стадией ликвидации (2011-2014) в материковой части Китая.
Результаты: В общей сложности в Китае в 2005-2014 гг. Было зарегистрировано 210 730 случаев малярии. Среднегодовая заболеваемость резко снизилась с 2,5 на 100 000 человек на этапе контроля до 0,2 на 100 000 на стадии ликвидации, но доля завозных случаев увеличилась с 9,8% до 41,0%. С начала Национальной программы ликвидации малярии в 2010 году общая доля лабораторно подтвержденных случаев последовательно увеличивалась, с самым высоким показателем 99,0% в 2014 г. Тем не менее, эта доля была значительно ниже в неэндемичных провинциях (79,0%), чем что в эндемичных провинциях (91,4%) в течение 2011-2014 гг. Средний интервал от начала болезни до постановки диагноза составила 3 дня на стадии ликвидации, при этом один день раньше, чем на этапе контроля. С 2011 года более

чем 99% случаев было зарегистрировано в течение 1 дня после постановки диагноза, в то время как доля случаев, которые были зарегистрированы в течение одного дня после установления диагноза была самой низкой в Тибете (37,5%). Сообщения о болезни сдвинулись из городских больниц на стадии контроля (67,9% случаев), в городские больницы и учреждения общественного здравоохранения на стадии ликвидации (69,4% случаев). Доля расследования в течение 3 дней улучшилась с 74,6% в 2010 году до 98,5% в 2014 году.
Заключение: Система наблюдения за индивидуальными малярии в Китае хорошо работает на стадии ликвидации малярии. Это гарантировало, малярия может быть диагностирована, информация о ней передана и своевременно исследована на местном уровне. Тем не менее, внутренние мигранты и зарубежные группы населения, а также случаи в исторически малярийных неэндемичных районах и труднодоступных областях являются новыми вызовами в проведении ликвидации малярии.

Translated from English version into Russian by Jekaterina Merkuljeva, through

Evaluación comparativa del diagnóstico, la notificación y la investigación de casos de malaria en China, 2005-2014: transición del control a la eliminación en el marco del programa nacional contra la malaria.

Jun-Ling Sun, Sheng Zhou, Qi-Bin Geng, Qian Zhang, Zi-Ke Zhang, Can-Jun Zheng, Wen-Biao Hu, Archie C.A. Clements, Sheng-Jie Lai and Zhong-Jie Li

Resumen
Introducción: Para eliminar la malaria se necesitan datos de vigilancia de alta calidad que permitan detectar y responder rápidamente a los casos individuales. La evaluación del desempeño de un sistema nacional de supervisión de malaria permitiría identificar deficiencias, que al ser abordadas, podrían mejorar el programa de vigilancia para la eliminación de la malaria.
Métodos: Se obtuvo información de casos correspondiente al periodo 2005-2014 del Sistema Nacional Chino de Información de Enfermedades Infecciosas de Notificación Obligatoria y del Sistema de Información de Vigilancia Intensiva Contra la Malaria. Se evaluó la incidencia de casos, la precisión y velocidad de diagnóstico. También se evaluaron informes e investigaciones, y se efectuaron comparaciones entre la fase de control de la enfermedad (2005-2010) y la fase de eliminación (2011-2014) en China continental.
Resultados: Se reportó un total de 210 730 casos de malaria en China continental entre 2005 y 2014. La incidencia anual promedio se redujo drásticamente, de 2,5 cada 100 000 personas en la fase de control a 0,2 cada 100 000 en la fase de eliminación. Sin embargo, la proporción de casos migratorios aumentó de un 9,8 % a un 41,0 %. Desde que se lanzó el Programa Nacional de Eliminación de Malaria en el 2010, mejoró considerablemente el porcentaje general de casos diagnosticados por pruebas de laboratorio, el cual alcanzó un pico máximo de 99,0% en el 2014. Sin embargo, esta proporción resultó ser mucho menor en las provincias donde la enfermedad no era endémica (79,0%) que en las provincias donde sílo era (91,4%) en el periodo 2011-2014. El intervalo medio entre el inicio de la enfermedad y el diagnóstico fue de tres días en la fase de eliminación, un día antes que en el intervalo de la fase de control. Desde 2011, el 99% de los casos fueron reportados dentro de un día luego de ser diagnosticados, mientras que la proporción de casos reportados dentro de un día luego de ser diagnosticados resulto ser la más baja en el Tíbet (37,5%). La fuente principal de notificación de casos pasó de ser hospitales municipales en la fase de control (67,9%) a ser hospitales a nivel ciudad e institutos de salud pública en la fase de eliminación (69,4% de los casos). La proporción de investigación dentro de los tres días luego de la notificación de caso mejoró, pasando de un 74,6% en 2010 a un 98,5% en 2014. .
Conclusiones: El sistema de vigilancia basado en casos individuales en China funcionó bien durante la fase de eliminación de malaria. Esto permitió diagnosticar los casos de malaria, notificarlos e investigarlos a tiempo a nivel local. Sin embargo, tanto los inmigrantes internos y las poblaciones extranjeras, como los casos en las áreas en las que históricamente la malaria no era endémica y en las zonas difíciles de alcanzar representan nuevos desafíos en lo que respecta a la vigilancia para eliminar la enfermedad.

Translated from English version into Spanish by Mpgorgone, through
